# Endothelial Differentiation of *CCM1* Knockout iPSCs Triggers the Establishment of a Specific Gene Expression Signature

**DOI:** 10.3390/ijms24043993

**Published:** 2023-02-16

**Authors:** Robin A. Pilz, Dariush Skowronek, Lara Mellinger, Sander Bekeschus, Ute Felbor, Matthias Rath

**Affiliations:** 1 Department of Human Genetics, University Medicine Greifswald and Interfaculty Institute of Genetics and Functional Genomics, University of Greifswald, 17475 Greifswald, Germany; 2 ZIK Plasmatis, Leibniz Institute for Plasma Science and Technology (INP), 17489 Greifswald, Germany; 3 Department of Human Medicine and Institute for Molecular Medicine, MSH Medical School Hamburg, 20457 Hamburg, Germany

**Keywords:** cerebral cavernous malformation, *CCM1/KRIT1*, CRISPR/Cas9 genome editing, iPSCs, iPSC-derived endothelial cells, RNA-Seq, endothelial-specific gene expression

## Abstract

Cerebral cavernous malformation (CCM) is a neurovascular disease that can lead to seizures and stroke-like symptoms. The familial form is caused by a heterozygous germline mutation in either the *CCM1*, *CCM2,* or *CCM3* gene. While the importance of a second-hit mechanism in CCM development is well established, it is still unclear whether it immediately triggers CCM development or whether additional external factors are required. We here used RNA sequencing to study differential gene expression in *CCM1* knockout induced pluripotent stem cells (*CCM1*^−/−^ iPSCs), early mesoderm progenitor cells (eMPCs), and endothelial-like cells (ECs). Notably, CRISPR/Cas9-mediated inactivation of *CCM1* led to hardly any gene expression differences in iPSCs and eMPCs. However, after differentiation into ECs, we found the significant deregulation of signaling pathways well known to be involved in CCM pathogenesis. These data suggest that a microenvironment of proangiogenic cytokines and growth factors can trigger the establishment of a characteristic gene expression signature upon *CCM1* inactivation. Consequently, *CCM1*^−/−^ precursor cells may exist that remain silent until entering the endothelial lineage. Collectively, not only downstream consequences of *CCM1* ablation but also supporting factors must be addressed in CCM therapy development.

## 1. Introduction

Cerebral cavernous malformations (CCMs) are capillary–venous lesions which are primarily found in the brain and spinal cord [1]. The familial form of this neurovascular disorder is inherited in an autosomal dominant manner with incomplete penetrance. Pathogenic variants in the *CCM1* gene (also known as *KRIT1*) can be identified in nearly 50% of patients with multiple CCMs or a positive family history [2]. Less frequently, germline mutations are detected in *CCM2* or *CCM3* (also known as *PDCD10)*. CCM lesions are associated with a significant risk of bleeding and can lead to headaches, seizures, paralysis, and other stroke-like symptoms [3]. The prevalence of symptomatic familial CCM cases is estimated to be about 1:5400 to 1:6200 [4]. One-third of the affected patients are minors, and no specific therapies are available yet [2].

A hallmark of CCM disease is pathological alterations of the endothelium. Endothelial dysfunction in general is characterized by comprehensive pathophysiological changes that include mechanisms such as pro-inflammatory and pro-thrombotic activation, impaired mechanotransduction, endothelial-to-mesenchymal transition, altered production of nitric oxide, or increased vascular permeability [5]. For a better understanding of the global molecular mechanisms in CCM pathogenesis, RNA sequencing analyses of endothelial cells derived from human CCM lesions [6,7], CCM-deficient animal models [7,8,9], and transient knockdown cell culture models [10,11,12] have been used to identify characteristic transcriptomic signatures. As a result, various deregulated signaling pathways have been described that contribute to the profound pathophysiological changes in CCM. Angiogenesis, cardiovascular development, cell-cell adhesion, cell-junction maintenance, apoptosis, hemostasis, and inflammation, for example, have all been identified to be highly deregulated in CCM models. Additionally, transcriptome analysis made it possible to identify differences between acute and chronic CCM models [8]. In the acute model, mostly cell proliferation-related genes were deregulated, while deregulation of inflammation-, permeability-, and adhesion-associated processes were seen in the chronic model.

It has long been established that a cellular recessive two-hit mechanism triggers CCM formation [13,14]. Although CCMs are benign vascular lesions, this mechanism is partly reminiscent of carcinogenesis. The identification of somatic *PIK3CA* mutations in human CCM tissues and the proof of clonal expansion of mutant endothelial cells in inducible CCM3 mouse models further support the concept that “cancer-like” mechanisms are involved in CCM pathogenesis [15,16,17]. The “cancer-like” behavior of mutant endothelial cells, however, is not limited to *CCM3* inactivation. Using blood outgrowth endothelial cells (BOECs), which are also referred to as endothelial colony-forming cells (ECFCs), we have previously shown that CRISPR/Cas9-induced *CCM1* ablation causes increased proliferation of mutant BOECs when co-cultured with wild-type cells [18].

Yet, it is still poorly understood whether there is a critical time window when CCM protein inactivation induces a CCM-specific phenotype. Interestingly, Malinverno and colleagues demonstrated that *Ccm3* inactivation in vessel-resident endothelial progenitor cells also led to CCM formation in mice [17]. Furthermore, non-genetic third hits are believed to play a critical role in CCM genesis [19]. To expand our knowledge on the role of *CCM1* in non-endothelial cell types and the importance of exogenous triggers in CCM formation, we combined our recently established isogenic *CCM1* knockout induced pluripotent stem cell (*CCM1*^−/−^ iPSC) model [20], the differentiation of iPSCs into endothelial-like cells (hereafter referred to as ECs), and RNA sequencing analyses. We demonstrate that the loss of *CCM1* expression leads to strong gene expression differences in ECs but not in iPSCs or early mesoderm progenitor cells (eMPCs). Our data suggest that a second somatic mutation followed by CCM1 inactivation does not necessarily result in a cellular or molecular phenotype but does lead to dramatic gene expression differences after endothelial differentiation.

## 2. Results

**Figure 1:**
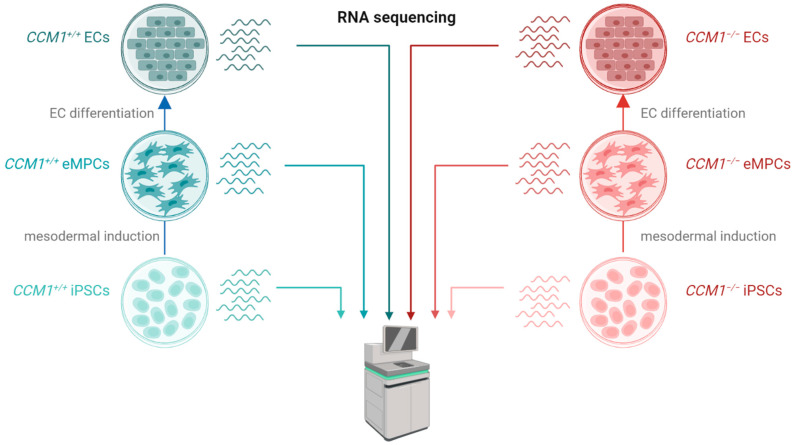
Experimental design of the study. *CCM1^+/+^* (blue) and *CCM1*^−/−^ (red) induced pluripotent stem cells (iPSCs) were differentiated into early mesoderm progenitor cells (eMPCs) and endothelial-like cells (ECs). RNA sequencing was used to study gene expression differences.

We have recently established a novel *CCM1* knockout iPSC model and demonstrated that *CCM1*^−/−^ iPSCs can be differentiated into ECs which show the upregulation of *KLF2* and *KLF4* as well as downregulation of *THBS1* [20]. These gene expression differences are well-characterized consequences of *CCM1* inactivation in ECs [9,21]. In our current study, we decided to analyze the gene expression profile in *CCM1*^−/−^ iPSC-derived ECs in more detail and to address the question whether CCM1 deficiency also leads to gene expression differences in iPSCs and eMPCs (Figure 1).

### 2.1. Major Gene Expression Differences in CCM1^−/−^ iPSC-Derived ECs

**Figure 2:**
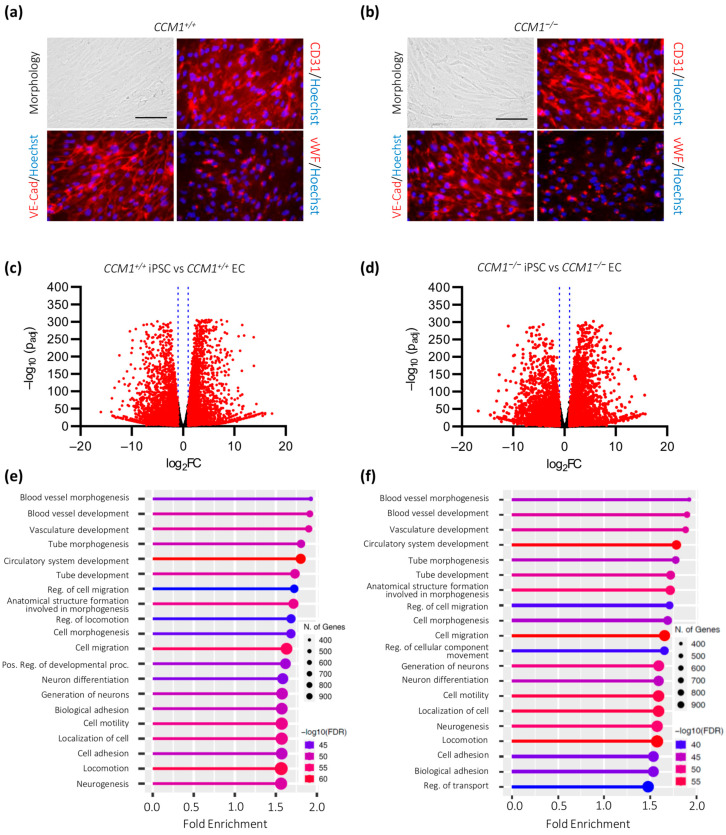
Targeted differentiation of iPSCs into ECs. (**a**,**b**) *CCM1^+/+^* (**a**) and *CCM1*^−/−^ (**b**) iPSC-derived ECs display typical EC morphology and express EC markers CD31, VE-Cadherin, and vWF (scale = 75 µm); (**c**,**d**) volcano plots of genes that are up- or downregulated upon differentiation of *CCM1^+/+^* (**c**) and *CCM1*^−/−^ (**d**) iPSCs into ECs. The log_2_ fold changes (log_2_FC) of the normalized mean hit counts are plotted against the negative log_10_ adjusted *p* values (−log_10_ (p_adj_)). Differentially expressed genes (=p_adj_ < 0.05 and |log_2_FC| > 1) are marked in red; (**e**,**f**) GO biological process enrichment analysis for *CCM1^+/+^* (**e**) and *CCM1*^−/−^ (**f**) cells. FDR = false discovery rate. The lollipop charts were created with ShinyGO 0.76 [22]. *n* = 3 per genotype.

Differentiation of *CCM1^+/+^* and *CCM1*^−/−^ iPSCs into ECs with the STEMdiff Endothelial Differentiation Kit (Figure 2a,b) led to the upregulation of 4470 and 4262 genes, respectively. At the same time, 4674 and 4468 genes were downregulated (Figure 2c,d). As expected, most of the differentially expressed genes could be assigned to angiogenesis-associated processes. No major alterations in differentiation-induced GO biological processes could be detected between the two genotypes (Figure 2e,f and Figure S1). Furthermore, mutant and wild-type cells showed a characteristic endothelial morphology and expressed the EC markers CD31, vWF, and VE-cadherin (Figure 2a,b). These results suggest that EC differentiation itself is not compromised by *CCM1* inactivation.

**Figure 3:**
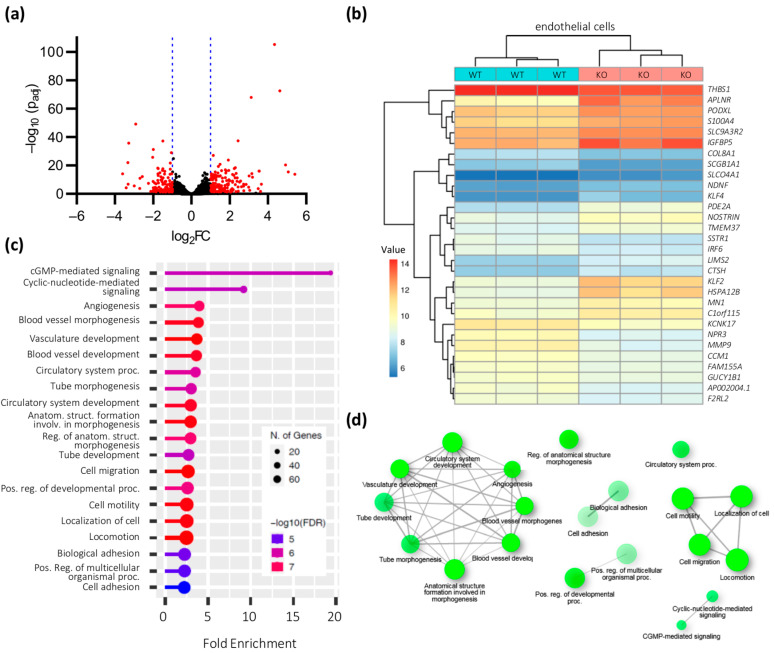
Gene expression differences in *CCM1*^−/−^ iPSC-derived ECs. (**a**) Volcano plot of up- or downregulated genes in *CCM1*^−/−^ iPSC-derived ECs. The log_2_ fold change (log_2_FC) of the normalized mean hit counts is plotted against the negative log_10_ adjusted *p* value (−log_10_ (p_adj_)). Differentially expressed genes (=p_adj_ < 0.05 and |log_2_FC| > 1) are marked in red; (**b**) heatmap of differentially expressed genes in *CCM1*^−/−^ iPSC-derived ECs. Shown are regularized log transformed read counts (= value) for *CCM1*^−/−^ (KO) and *CCM1^+/+^* (WT) iPSC-derived ECs; (**c**) GO biological process enrichment analysis for differentially expressed genes in *CCM1*^−/−^ iPSC-derived ECs. To avoid a systemic bias, *CCM1* was excluded from the enrichment analysis. FDR = false discovery rate; (**d**) enriched GO terms are visualized as a network. The size of the network nodes reflects the number of genes. Lines connect related terms. The thickness of the lines reflects the percent of overlapping genes. The lollipop chart (**c**) and the network (**d**) were created with ShinyGO 0.76 [22]. *n* = 3 per genotype.

When we compared the gene expression profiles of *CCM1^+/+^* and *CCM1*^−/−^ iPSC-derived ECs, we found major differences: 181 and 161 genes were significantly up- or downregulated in *CCM1*^−/−^ ECs, respectively (Figure 3a). Among the most upregulated genes were *LHX6* (LIM Homeobox 6; log_2_FC = 5.4), *CMKLR1* (Chemerin Chemokine-Like Receptor 1; log_2_FC = 5.1), *APLNR* (Apelin Receptor; log_2_FC = 4.9), *HSPA12B* (Heat Shock Protein Family A (Hsp70) Member 12B; log_2_FC = 4.6), and *KLF2* (Krueppel-Like Factor 2; log_2_FC = 4.3). The top 10 upregulated genes also included *KLF4* (Krueppel-Like Factor 4; log_2_FC = 3.5). Less highly upregulated was, for example, *THBD* (Thrombomodulin; log_2_FC = 1.1). Strongly downregulated, on the other hand, were *TCN1* (Transcobalamin 1; log_2_FC = −3.6), *MT1G* (Metallothionein 1G; log_2_FC = −3.4), *SCGB1A1* (Secretoglobin Family 1A Member 1; log_2_FC = −3.3), *CRABP1* (Cellular Retinoic Acid Binding Protein 1; log_2_FC = −3.3), and *MMP9* (Matrix Metallopeptidase 9; log_2_FC = −3.3) (Figure 3b).

The deregulated genes could be assigned to different clusters of biological processes. As expected, angiogenesis-associated and vessel formation-related processes were among them. In addition, genes involved in cell motility were also deregulated (Figure 3c,d). Analysis with the KEGG Mapper Search tool also revealed the deregulation of several CCM-associated pathways [23,24] such as the regulation of actin cytoskeleton, Notch signaling, ECM-receptor interaction, or cytokine–cytokine receptor interaction (Table S1). Following up on this, we also performed protein analyses and used an antibody array for screening of several angiogenesis-related proteins (Figure S2, Table S2), as well as bead-based quantification of selected inflammation-associated targets (Figure S3). However, we did not observe major differences. Given that we have only examined a limited number of targets and that certain processes in CCM formation such as inflammatory mechanisms may only be activated under in vivo or co-culture conditions [25,26], further studies are needed to elucidate the proteomic effect of *CCM1* knockout. 

**Figure 4:**
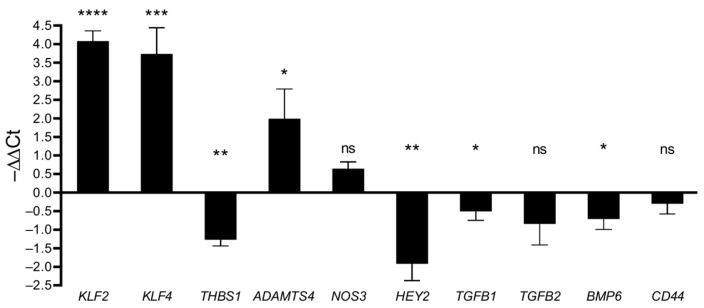
RT-qPCR analyses of selected transcripts in *CCM1*^−/−^ ECs compared to *CCM1^+/+^* ECs (*n* = 3 per genotype). For statistical analyses, student’s two-tailed *t* tests were used: * *p* < 0.05, ** *p* < 0.01, *** *p* < 0.001, **** *p* < 0.0001, ns = not significant.

Next, we investigated the gene expression differences of several targets known to be involved in CCM pathogenesis and used reverse transcription quantitative PCR (RT-qPCR) for validation analyses (Figure 4 and Figure S4, Table S3). Especially, MEKK3-KLF2/4 signaling is considered a central mechanism that regulates multiple signaling pathways associated with endothelial dysfunction (Figure S5) [27]. Besides the strong upregulation of *KLF2* and *KLF4* in *CCM1*^−/−^ ECs, we observed altered expression levels of several KLF2/4-regulated genes that are known to play a role in CCM disease. This includes the downregulation of *THBS1* (TSP1, Thrombospondin 1, log_2_FC = −1.1) and upregulation of *ADAMTS4* (ADAM Metallopeptidase with Thrombospondin Type 1 Motif 4, log_2_FC = 2.2). We did not see major expression differences (namely |∆∆Ct| > 1 and *p* < 0.05) for *TGFB1*, *TGFB2*, *BMP6*, *CD44*, and *NOS3* (Figure 4 and Figures S2–S4) which would have been indicative of endothelial-to-mesenchymal transition or altered expression of endothelial nitric oxide synthase (eNOS) [28,29,30]. However, other known effects of *CCM1* inactivation in ECs, namely, decreased expression of the NOTCH target gene *HEY2* (Hes Related Family BHLH Transcription Factor with YRPW Motif 2, log_2_FC = −1.7), could be recapitulated in our system. Collectively, these data support the validity of this new model.

### 2.2. Minor Gene Expression Differences in CCM1^−/−^ iPSC and CCM1^−/−^ iPSC-Derived eMPCs

**Figure 5:**
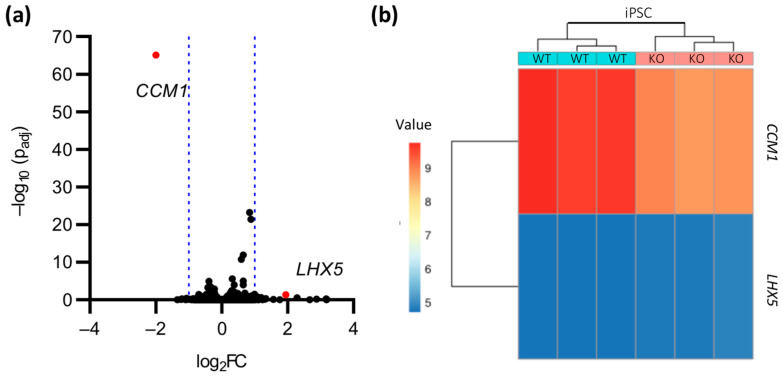
Gene expression differences in *CCM1*^−/−^ iPSCs. (**a**) Volcano plot of up- or downregulated genes in *CCM1*^−/−^ iPSCs. The log_2_ fold change (log_2_FC) of the normalized mean hit counts is plotted against the negative log_10_ adjusted *p* value (−log_10_ (p_adj_)). Differentially expressed genes (= p_adj_ < 0.05 and |log_2_FC| > 1) are marked in red; (**b**) heatmap of differentially expressed genes in *CCM1*^−/−^ iPSCs. Shown are regularized log transformed read counts (= value) for *CCM1*^−/−^ (KO) and *CCM1^+/+^* (WT) iPSCs. *n* = 3 per genotype.

We next asked whether *CCM1* inactivation also leads to a characteristic molecular phenotype in iPSCs and subjected *CCM1^+/+^* and *CCM1*^−/−^ iPSCs, which showed typical iPSC morphology and expression of pluripotency markers SSEA4, OCT4, SOX2, and TRA-1-60 (Figure S6a), to RNA-seq analysis. However, we could find hardly any gene expression differences (Figure 5a,b). Besides the downregulation of *CCM1* gene expression (log_2_FC = −2.0), only *LHX5* was significantly upregulated (LIM Homeobox 5; log_2_FC = 1.9). Yet, the adjusted *p* value for *LHX5* was only slightly below the significance level (p_adj_ = 0.045). These data suggest that *CCM1* expression is dispensable in iPSCs and that its inactivation does not result in a molecular phenotype. 

**Figure 6:**
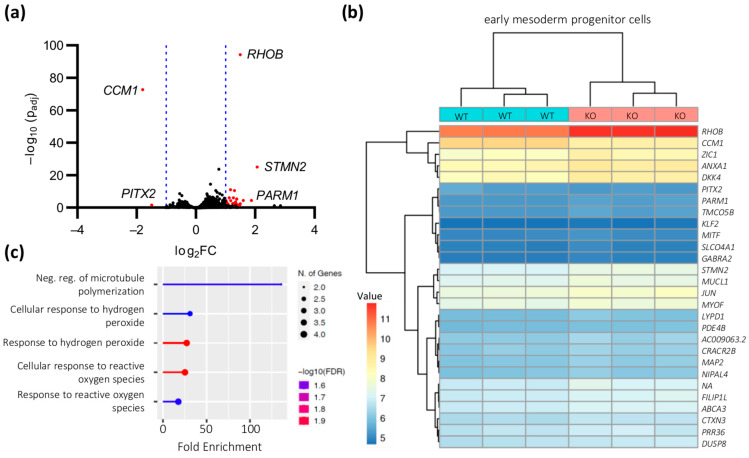
Gene expression differences in *CCM1*^−/−^ iPSC-derived eMPCs. (**a**) Volcano plot of up- or downregulated genes in *CCM1*^−/−^ iPSC-derived eMPCs. The log_2_ fold change (log_2_FC) of the normalized mean hit counts is plotted against the negative log_10_ adjusted *p* value (−log_10_ (p_adj_)). Differentially expressed genes (=p_adj_ < 0.05 and |log_2_FC| > 1) are marked in red; (**b**) heatmap of differentially expressed genes in *CCM1*^−/−^ iPSC-derived eMPCs. Shown are regularized log transformed read counts (=value) for *CCM1*^−/−^ (KO) and *CCM1^+/+^* (WT) iPSC-derived eMPCs; (**c**) GO biological process enrichment analysis for differentially expressed genes in *CCM1*^−/−^ iPSC-derived eMPCs. To avoid a systemic bias, *CCM1* was excluded from the enrichment analysis. FDR = false discovery rate. The lollipop chart was created with ShinyGO 0.76 [22]. *n* = 3 per genotype.

An intermediate stage of differentiation from iPSCs to ECs with the STEMdiff Endothelial Differentiation Kit are eMPCs. After having verified the expression of the mesoderm markers α-SMA and Brachyury (Figure S6b), we also profiled gene expression levels for these cells by RNA-seq to characterize the effects of CCM1 inactivation in more detail. Although we detected slightly stronger differences in these cells than in iPSCs, only 26 genes were upregulated and 2 genes were downregulated (Figure 6a). As expected, one of these genes was *CCM1* (log_2_FC = −1.8). Other deregulated genes included: *STMN2* (Stathmin 2; log_2_FC = 2.1), *PARM1* (Prostate Androgen-Regulated Mucin-Like Protein 1; log_2_FC = 1.9), *LYPD1* (LY6/PLAUR Domain Containing 1; log_2_FC = 1.6), *RHOB* (Ras Homolog Family Member B; log_2_FC = 1.5), *SLCO4A1* (Solute Carrier Organic Anion Transporter Family Member 4A1; log_2_FC = 1.5), and *KLF2* (Krueppel-Like Factor 2; log_2_FC = 1.4) (Figure 6b and Figure S4). Our biological process enrichment analysis found only a moderate enrichment for the negative regulation of microtubule polymerization and reactive oxygen species-related clusters (Figure 6c). However, we also found only moderate significance levels for eMPCs.

To put our findings on gene expression differences between *CCM1^+/+^* and *CCM1*^−/−^ iPSCs, eMPCs, and ECs in a broader context, we additionally performed principal component analysis (PCA) for dimensionality reduction. PCA for all RNA-seq samples resulted in three highly distinguishable groups comprised of only iPSC, eMPC, or EC samples, emphasizing the big gap in gene expression between each step of differentiation (Figure S7a). Next, we performed PCA for all cell types individually (Figure S7b–d). In line with our other data, PCA revealed the smallest differences between *CCM1^+/+^* and *CCM1*^−/−^ iPSCs, while the biggest differences occur after differentiation into the endothelial lineage.

## 3. Discussion

The results of our current study presenting comprehensive RNA sequencing data from *CCM1*^−/−^ iPSCs, eMPCs, and ECs allow two conclusions: (1) CCM1 is dispensable for EC differentiation but essential for the maintenance of endothelial quiescence and (2) CCM1 apparently does not play a critical role in iPSCs and eMPCs.

Upon the induction of endothelial differentiation, *CCM1*^−/−^ iPSCs significantly activated signaling cascades related to blood vessel development and morphogenesis, tube formation, and cell migration. Furthermore, *CCM1*^−/−^ iPSC-derived ECs showed normal morphology and expressed typical EC markers. However, when we compared *CCM1*^−/−^ and *CCM1^+/+^* iPSC-derived ECs on the molecular level, we found hundreds of differentially expressed genes. Among the most upregulated transcripts in mutant ECs were *KLF2* and *KLF4*, which are crucial mediators of profound dysfunction in ECs lacking CCM1, CCM2, or CCM3 [21,31,32,33]. Interestingly, analyses in hCMEC/D3 cells (human cerebral microvascular endothelial cell line), HUVECs (human umbilical vein endothelial cells), and zebrafish have recently demonstrated that not only genetic *CCM1* inactivation but also the inhibition of the HEG1 (heart of glass 1)-CCM1 protein complex can induce upregulation of KLF2 and KLF4 levels in ECs [34]. However, irrespective of its causes, a gain of MEKK3-KLF2/4 signaling in ECs can trigger mechanisms that contribute to CCM formation, progression, and hemorrhage. Our RNA-seq data show that the deregulation of several important CCM-associated biological processes, including altered expression of *THBS1* [9], *THBD* [35], *ADAMTS4* [21], or *HEY2* [12], could be recapitulated in differentiated *CCM1*^−/−^ ECs. Although future studies will have to substantiate endothelial dysfunction at the protein level, it is evident from our data that the inactivation of *CCM1* in ECs apparently cannot be compensated and disrupts endothelial quiescence. Our RNA sequencing analysis of *CCM1*^−/−^ iPSCs, eMPCs, and ECs can obviously not exclude molecular consequences of *CCM1* inactivation in other cell types. Indeed, for the *CCM3* gene, it has been reported that its inactivation in neuroglia and mural cells induces CCM-like lesions in mice [36,37]. However, our results suggest that there might be *CCM1*^−/−^ precursor cells which remain silent until they come into a specific microenvironment and differentiate into endothelial cells. 

**Figure 7:**
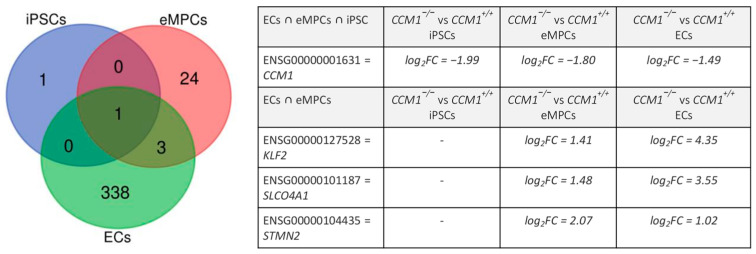
Overlap of differentially expressed genes in iPSCs, eMPCs, and ECs. Differential gene expression was defined as p_adj_ < 0.05 and |log_2_FC| > 1. iPSCs = *CCM1*^−/−^ vs. *CCM1^+/+^* iPSCs; eMPCs = *CCM1*^−/−^ vs. *CCM1^+/+^* eMPCs; ECs = *CCM1*^−/−^ vs. *CCM1^+/+^* ECs.

Although CCM1 is ubiquitously expressed in early embryogenesis and found with high expression levels in various cell lines [38,39,40], its function in non-endothelial cell types remains poorly understood. Consistent with the assumption that intact CCM function is required specifically in ECs [27], we found very few gene expression differences in *CCM1* knockout eMPCs and iPSCs. In addition, the overlap of up- or downregulated genes in *CCM1*^−/−^ ECs, eMPCs, and iPSCs was very small. Apart from the CRISPR/Cas9-induced disruption of *CCM1* gene expression, there was no overlap between iPSCs and eMPCs or ECs, largely because only two genes were differentially expressed in *CCM1*^−/−^ iPSCs anyway. However, there was also little overlap between eMPCs and ECs (Figure 7). 

Although *KLF2* was also moderately upregulated in *CCM1*^−/−^ eMPCs, the expression difference increased significantly when the cells were further differentiated into ECs. The same is true for *SLCO4A1* whose role in CCM pathogenesis is unclear so far. In contrast, the expression levels of *KLF4* and nearly all other genes that were up- or downregulated in mutant ECs were unchanged in *CCM1*^−/−^ eMPCs or iPSCs. These results suggest that the establishment of a *CCM1* knockout-specific gene expression pattern is enabled by exogenous factors such as cytokines and growth factors used to induce EC differentiation. The idea that the multiple downstream effects of *CCM* gene inactivation may occur only in a specific microenvironment is not completely new. In 2011, the groups of Elisabetta Dejana and Elisabeth Tournier-Lasserve reported that EC-specific ablation of *Ccm2* in mice only led to a CCM phenotype when induced in developmental stages of active angiogenesis [41]. Furthermore, the inhibition of VEGF signaling, which is essential for angiogenesis in vivo and also for the differentiation of iPSCs into ECs, was shown to reduce the number of CCM lesions in *Ccm1* induced endothelial cell knockout mice (*Krit1^ieKO^*) [42,43]. This also reinforces the important role of exogenous triggers for CCM formation. Even though additional factors occurring in vivo, e.g., cell-cell interactions with pericytes and astrocytes [30,44] or ECs with preserved CCM expression [25], are most likely also necessary to trigger the comprehensive pathophysiological changes after CCM protein loss, our study strengthens that a specific microenvironment is one important factor in the multi-step pathomechanism in CCM disease. 

Collectively, our results suggest that in our search for new CCM therapies, we should address not only the consequences of CCM ablation but also the supporting factors for endothelial dysfunction.

## 4. Materials and Methods

### 4.1. Cell Culture of Induced Pluripotent Stem Cells (iPSCs) 

The *CCM1*^+/+^ and *CCM1*^−/−^ iPSC lines used in this study were derived from the parental AICS-0023 iPSC line (Allen Cell Collection, Coriell Institute, Camden, NJ, USA) before [20]. IPSCs were maintained in Essential 8 Flex medium (Thermo Fisher Scientific, Waltham, MA, USA) at 37 °C and 5% CO_2_ on plates coated with growth factor reduced Matrigel (Corning, New York, NY, USA). Cultures were routinely passaged with 0.5 mM EDTA (Thermo Fisher Scientific). 

### 4.2. CRISPR/Cas9 Editing and Single-Cell Cloning

The CRISPR/Cas9 genome editing protocol to generate the *CCM1*^−/−^ iPSC lines used in this study was already described before [20]. In brief, AICS-0023 iPSCs were transfected with single guide RNA (sgRNA):Cas9 ribonucleoprotein complexes with the target region located in exon 10 of *CCM1* (LRG_650t1, 5′-GGAGCTCCTAGACCAAAGTA-3′; Integrated DNA Technologies, Coralville, IA, USA) using Lipofectamine Stem Transfection Reagent (Thermo Fisher Scientific). IPSCs were reverse transfected following detachment with StemPro Accutase (Thermo Fisher Scientific) on growth factor reduced Matrigel-coated 24-well plates. For single-cell cloning, statistically 0.5 cells/well were plated on 96-well plates in Essential 8 Flex medium supplemented with CloneR (STEMCELL Technologies, Vancouver, BC, Canada). Genomic DNA of expanded cells was isolated and sequenced by Sanger sequencing.

### 4.3. Differentiation of iPSCs into eMPCs and ECs

The STEMdiff Endothelial Differentiation Kit (STEMCELL Technologies) was used according to the manufacturer’s instructions for the differentiation of iPSCs into eMPCs and ECs. Initially, iPSCs were detached with StemPro Accutase (Thermo Fisher Scientific) and seeded at a density of 100,000 cells per 6-well in Essential 8 Flex medium supplemented with 10 µM ROCK inhibitor Y-27632 (STEMCELL Technologies). On day 7, differentiated ECs were subcultured at a density of 150,000 cells per 6-well. The cells were expanded in STEMdiff Endothelial Expansion Medium (STEMCELL Technologies) for four days, then split on passage #1, and cultured for an additional four days. RNA was extracted for eMPCs on day 3 of the differentiation protocol and at passage #1 for expanded differentiated ECs.

The PSC 4-Marker Immunocytochemistry Kit (Thermo Fisher Scientific) was used to analyze SSEA4, OCT4, SOX2, and TRA-1-60 expression for iPSCs. An anti-mouse Alexa Fluor 555 antibody (ab150114, 1:500, Abcam, Cambridge, UK) and an anti-rat Alexa Fluor 555 antibody (A-21434, 1:500, Thermo Fisher Scientific) were used as alternative secondary antibodies for SSEA4 and SOX2 staining. Unless otherwise specified, the Immunofluorescence Application Solutions Kit (Cell Signaling, Danvers, MA, USA) was used according to the manufacturer’s instructions for marker staining after the differentiation of iPSCs into eMPCs and ECs. For eMPCs, immunofluorescence analysis for α-SMA (ab7817, 1:100, Abcam) and Brachyury (AF2085, 1:20, Bio-Techne, Minneapolis, MN, USA) was performed. For Brachyury staining, cells were permeabilized and blocked in 1% bovine serum albumin, 0.3% Triton X-100, and 10% normal donkey serum. For ECs, endothelial marker expression at passage #1 was evaluated by the staining of CD31 (3528S, 1:800, Cell Signaling), VE-Cadherin (2500S, 1:400, Cell Signaling), and VWF (MA5-14029, 1:66, Thermo Fisher Scientific). An anti-mouse Alexa Fluor 555 antibody (ab150114, 1:200, Abcam), an anti-rabbit Alexa Fluor 555 antibody (A-21429, 1:500, Thermo Fisher Scientific), and an anti-goat Alexa Fluor 555 antibody (ab150130, 1:200, Abcam) were used as secondary antibodies. DAPI or Hoechst 33342 (Thermo Fisher Scientific) were used for nuclei staining.

### 4.4. RNA Sequencing and Data Analysis

The Direct-zol RNA MiniPrep Plus Kit (Zymo Research, Irvine, CA, USA) was used for RNA purification. Concentration measurements and sample integrity control were performed with a Qubit 4.0 fluorometer (Thermo Fisher Scientific) and on a 2100 Bioanalyzer G2939AA instrument (Agilent, Santa Clara, CA, USA), respectively. RNA library preparation by polyA selection and sequencing on an Illumina NovaSeq (Illumina, San Diego, CA, USA) with 2 × 150 cycles was done by Azenta Life Sciences (Leipzig, Germany). Trimming of reads and mapping to the human reference genome assembly GRCh37 was performed with Trimmomatic v.0.36 and STAR aligner v.2.5.2b. Gene hit counts were extracted with featureCounts from the Subread package v.1.5.2. Principal component analysis of RNA sequencing data was performed with pcaExplorer [45,46]. The 300 genes with the highest inter-sample variance were used to compute the principal components. The confidence ellipses were created with a confidence interval level of 0.95. For RT-qPCR validation analyses, RNA was reverse transcribed into cDNA using the First Strand cDNA Synthesis Kit (Thermo Fisher Scientific). SYBR Green-based qPCR analyses were performed on a Roche Light Cycler 480 instrument (Roche, Basel, Switzerland) to determine relative gene expression levels using *RPLP0* as an endogenous control. The primer sequences are given in Table S3.

### 4.5. Protein Assays

For the expression analysis of angiogenesis-related proteins in differentiated ECs, 250 µg of protein were used per sample to perform the Human Angiogenesis Antibody Array (ab134000, Abcam) according to the manufacturer’s instructions. Protein was harvested with RIPA Lysis and Extraction Buffer (Thermo Fisher Scientific) supplemented with Halt Protease and Phosphatase Inhibitor Cocktail (Thermo Fisher Scientific) according to the manufacturer’s instructions. Protein concentrations were determined with the Micro BCA Protein Assay Kit (Thermo Fisher Scientific). The Protein Array Analyzer for the ImageJ 1.53t software [47] was used to determine spot intensities on membranes.

To measure the concentration of signaling molecules in cell culture supernatants of differentiated ECs, cells were cultivated for three days and supernatants were collected and frozen. Cell culture supernatants were subjected to multiplex chemokine, cytokine, and growth factor analysis using the LEGENDplex assay (BioLegend, Amsterdam, The Netherlands) according to the manufacturer’s instructions. Briefly, samples were incubated with capture beads with fluorescent barcodes and coated with monoclonal antibodies targeting different analytes. After washing, the beads were incubated with phycoerythrin (PE)-conjugated monoclonal antibodies specific for another epitope of each analyte tested. Subsequent to additional washes, samples were acquired by flow cytometry (CytoFLEX LX, Beckman-Coulter, Krefeld, Germany). The PE intensities informed about absolute analyte quantities by interpolation from 5-log analyte standards measured in parallel.

### 4.6. Statistical Analysis

DESeq2 was used for the analysis of differential gene expression in RNA-seq data. To generate *p* values and log_2_ fold changes, the Wald test was utilized. Genes with an adjusted *p* value of <0.05 and an absolute log_2_ fold change > 1 were considered differentially expressed between the compared groups. Gene ontology analysis was performed with ShinyGO 0.76.1 [22] and an FDR cut-off of 0.05. For the RT-qPCR validation analyses and concentration measurements of signaling molecules, student’s two-tailed *t* tests were used.

## Data Availability

RNA sequencing data were uploaded to the Gene Expression Omnibus (GEO) database (record number: GSE214306). All other relevant data are published within the paper.

## Supplementary Materials

The following supporting information can be downloaded at: https://www.mdpi.com/article/10.3390/ijms24043993/s1.
