# Supplementary material for: Endothelial Differentiation of CCM1 Knockout iPSCs Triggers the Establishment of a Specific Gene Expression Signature
Source: Int J Mol Sci. 2023 Feb 16;24(4):3993. doi: 10.3390/ijms24043993 (PMC9963194; doi:10.3390/ijms24043993)
Supplement: Supplementary file 1 [file ijms-24-03993-s001.zip › ijms-2029230 - Supplementary Figures.pdf]

Supplementary Figures

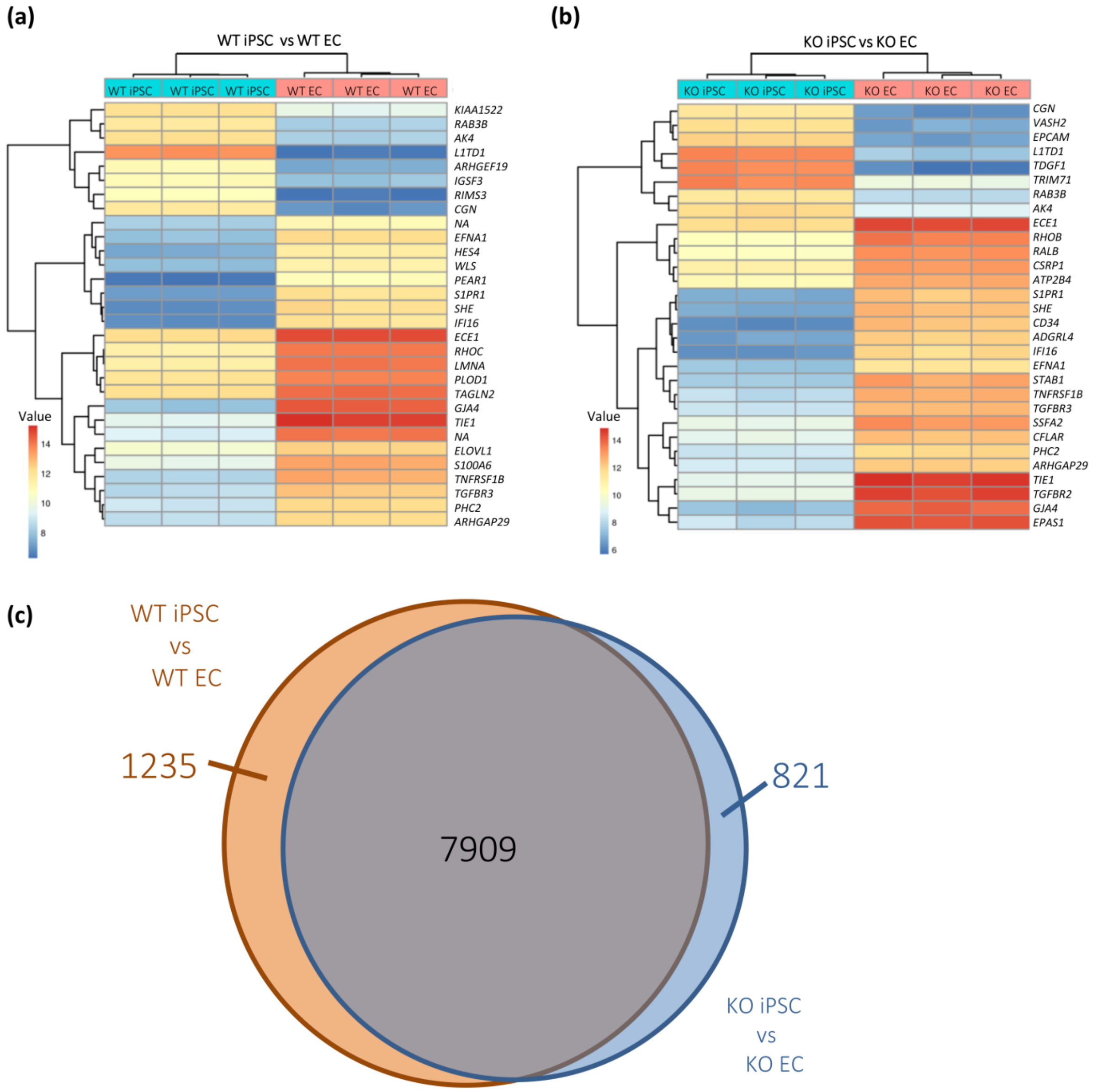

Figure S1. Heatmaps of significantly up- or downregulated genes in targeted differentiation of *CCM1*$^{+/+}$ (**a**) and *CCM1*$^{-/-}$ (**b**) iPSCs into ECs. Shown are regularized log transformed read counts for cells. Differentially expressed genes = $p_{adj} < 0.05$ and $|\log_2FC| > 1$. $n = 3$ per genotype. WT = *CCM1*$^{+/+}$ wild-type cells; KO = *CCM1*$^{-/-}$ knockout cells. (**c**) Venn diagram of the overlap of differentially expressed genes in both conditions.

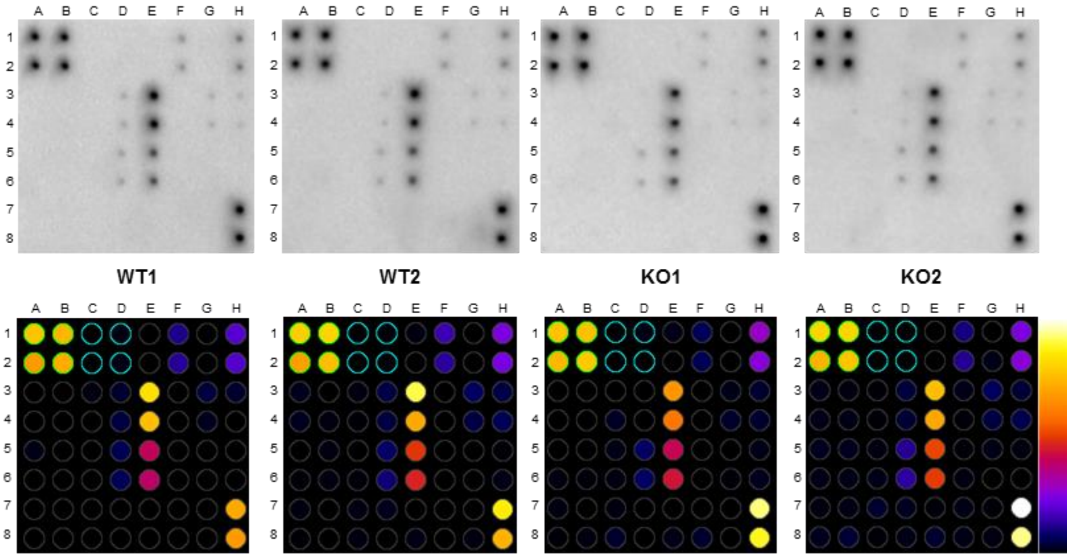

Figure S2. Human angiogenesis antibody array of iPSC-derived *CCM1*$^{+/+}$ (WT) and *CCM1*$^{-/-}$ (KO) ECs. The array was performed for two WT and two KO samples. Two spots per angiogenic factor are located on the membrane. The top row shows raw membrane images. The bottom row shows normalized spot intensities calculated with the Protein Array Analyzer for ImageJ. Samples were normalized to WT1. The positive control spots A1, A2, B1, B2 and the negative control spots C1, C2, D1, D2 were used as references. Positive controls = A1,A2,B1,B2,H7,H8; negative controls = C1,C2,D1,D2,G7,G8; GRO = A3,A4; PIGF = A5,A6; IFN-γ = B3,B4; RANTES = B5,B6; IGF-I = C3,C4; TGF-β1 = C5,C6; IL-6 = D3,D4; TIMP-1 = D5,D6; Angiogenin = E1,E2; IL-8 = E3,E4; TIMP-2 = E5,E6; EGF = F1,F2; LEPTIN = F3,F4; Thrombopoietin = F5,F6; ENA-78 = G1,G2; MCP-1 = G3,G4; VEGF = G5,G6; bFGF = H1,H2; PDGF-BB = H3,H4; VEGF-D = H5,H6. Remaining positions are blank.

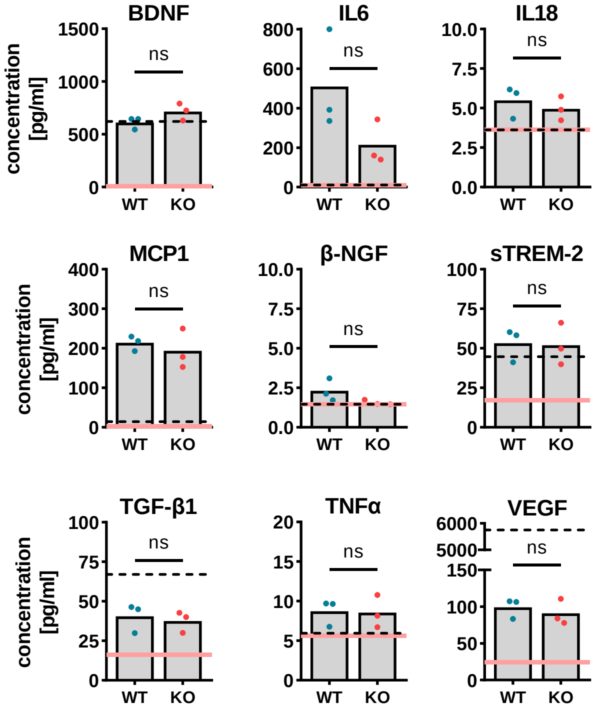

Figure S3. Concentrations of selected signaling molecules in cell culture supernatants of iPSC-derived *CCM1*$^{+/+}$ (WT) and *CCM1*$^{-/-}$ (KO) ECs (n = 3 per genotype). The detection limit of the assay is shown as a pink line. Cell culture medium served as a control (black dotted line). For statistical analyses, student's two-tailed t tests were used. ns = not significant.

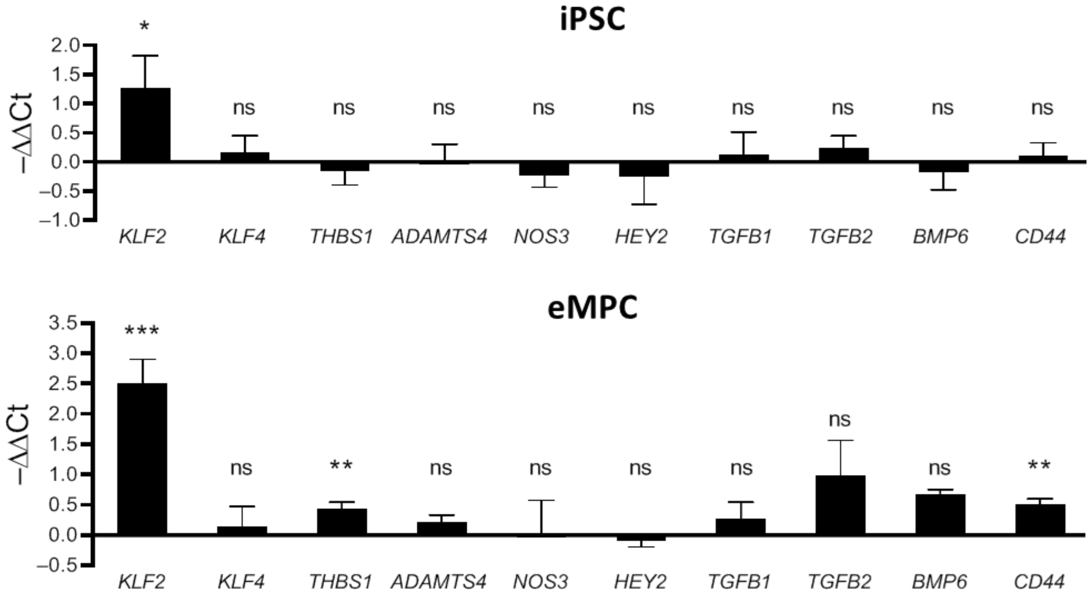

Figure S4. RT-qPCR analyses of selected transcripts in *CCM1*$^{-/-}$ iPSCs and eMPCs compared to *CCM1*$^{+/+}$ condition ($n = 3$ per genotype each). For statistical analyses, student's two-tailed t tests were used: * $p < 0.05$, ** $p < 0.01$, *** $p < 0.001$, ns = not significant.

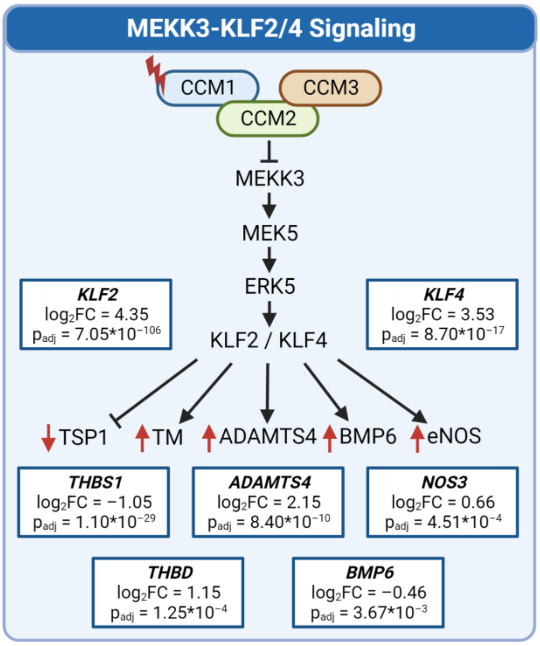

Figure S5. MEKK3-KLF2/4 signaling in CCM disease. Red arrows symbolize known expression changes of KLF2/4-regulated targets after CCM1 inactivation. Gene expression changes in differentiated *CCM1*$^{-/-}$ ECs as determined in this study are shown in the boxes.

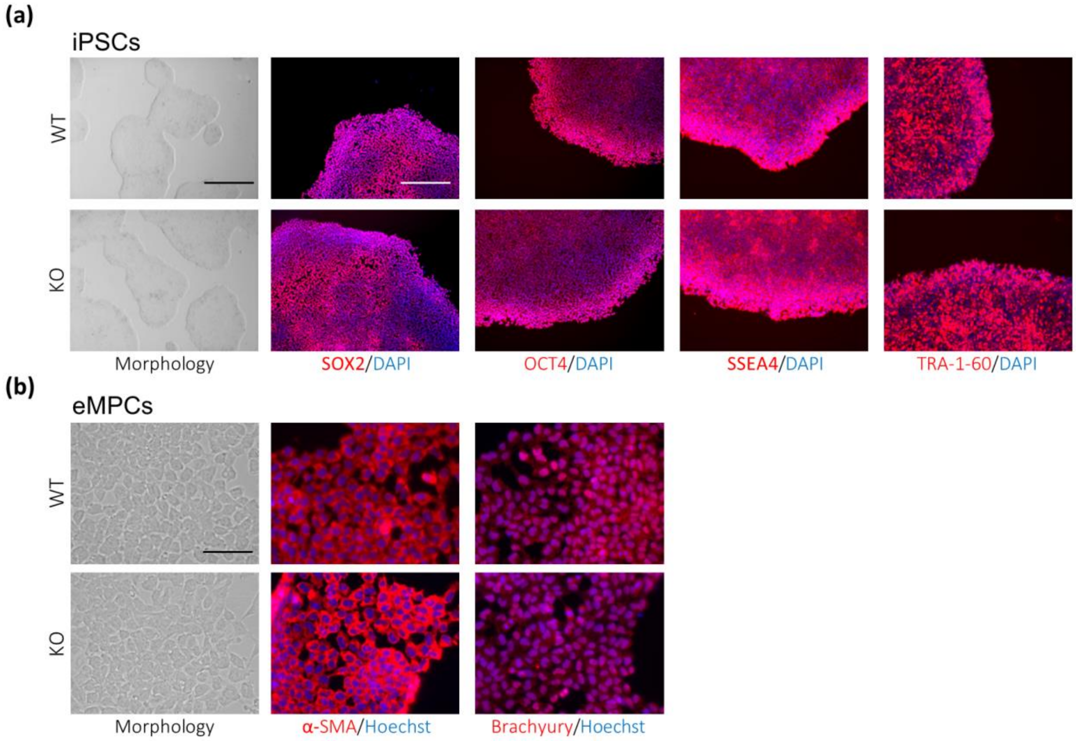

Figure S6. Expression of cell type specific markers in *CCM1*$^{+/+}$ (WT) and *CCM1*$^{-/-}$ (KO) cells. **(a)** Representative images of the iPSC morphology (scale = 1000 μm) and the expression of the stem cell markers SOX2, OCT4, SSEA4, and TRA-1-60 (scale = 400 μm). **(b)** Representative images of the eMPC morphology and the expression of mesoderm markers α-SMA and Brachyury (scale = 75 μm).

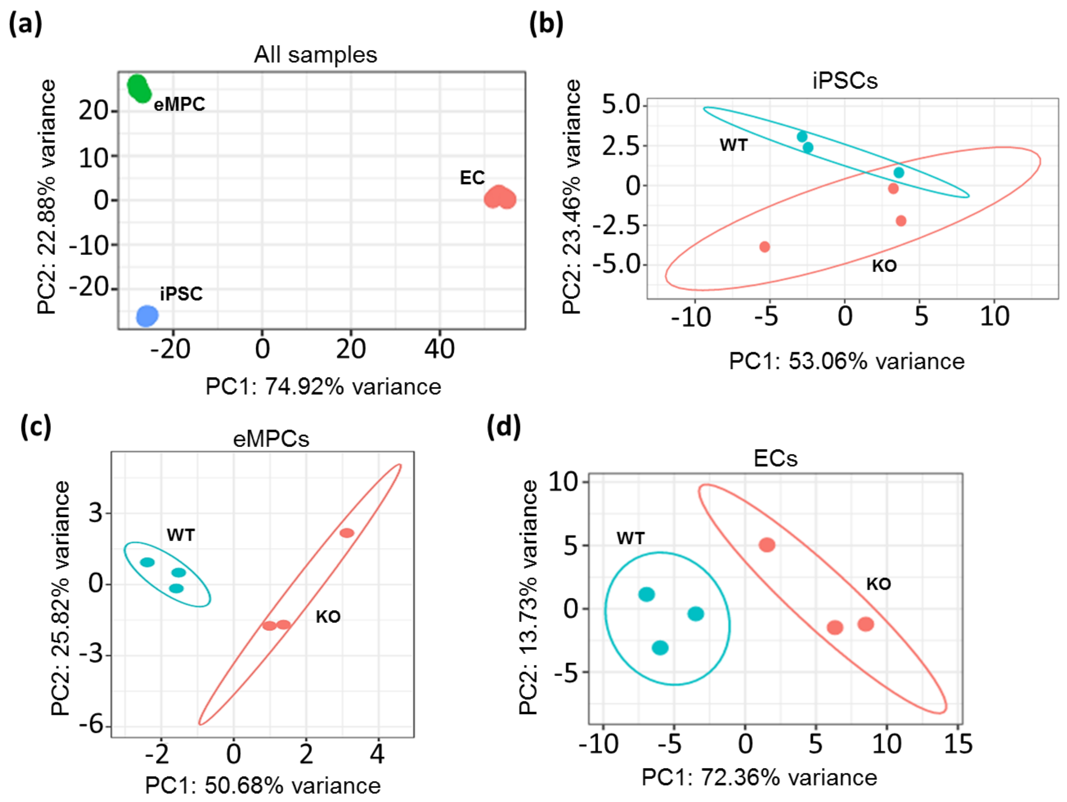

Figure S7. Principal component analysis (PCA) of RNA sequencing data of *CCM1*$^{+/+}$ (WT) and *CCM1*$^{-/-}$ (KO) iPSCs, eMPCs, and ECs. **(a)** PCA of all samples subjected to RNA sequencing revealed large differences between iPSCs, eMPCs, and ECs. **(b)** For iPSC samples, PCA shows close proximity of WT and KO cells. **(c,d)** For eMPCs and ECs, two distinct populations of WT and KO cells can be observed. The largest differences between KO and WT cells can be observed in ECs. Here, the principal component (PC) 1 represents 72.36% of variance between samples. PcaExplorer was used to perform the analyses.
